# The Association of a Geographically Wide Social Media Network on Depression: County-Level Ecological Analysis

**DOI:** 10.2196/43623

**Published:** 2023-03-27

**Authors:** Alaina M Beauchamp, Christoph U Lehmann, Richard J Medford, Amy E Hughes

**Affiliations:** 1 Department of Epidemiology, Human Genetics, and Environmental Sciences School of Public Health University of Texas Health Science Center at Houston Dallas, TX United States; 2 Peter O'Donnell Jr School of Public Health University of Texas Southwestern Medical Center Dallas, TX United States; 3 Department of Pediatrics University of Texas Southwestern Medical Center Dallas, TX United States; 4 Lyda Hill Department of Bioinformatics University of Texas Southwestern Medical Center Dallas, TX United States; 5 Clinical Informatics Center University of Texas Southwestern Medical Center Dallas, TX United States; 6 Department of Internal Medicine University of Texas Southwestern Medical Center Dallas, TX United States

**Keywords:** Facebook, social connectedness, depression, county-level analysis, social media, mental health, research, ecological, geography, GIS

## Abstract

**Background:**

Social connectedness decreases human mortality, improves cancer survival, cardiovascular health, and body mass, results in better-controlled glucose levels, and strengthens mental health. However, few public health studies have leveraged large social media data sets to classify user network structure and geographic reach rather than the sole use of social media platforms.

**Objective:**

The objective of this study was to determine the association between population-level digital social connectedness and reach and depression in the population across geographies of the United States.

**Methods:**

Our study used an ecological assessment of aggregated, cross-sectional population measures of social connectedness, and self-reported depression across all counties in the United States. This study included all 3142 counties in the contiguous United States. We used measures obtained between 2018 and 2020 for adult residents in the study area. The study’s main exposure of interest is the Social Connectedness Index (SCI), a pair-wise composite index describing the “strength of connectedness between 2 geographic areas as represented by Facebook friendship ties.” This measure describes the density and geographical reach of average county residents’ social network using Facebook friendships and can differentiate between local and long-distance Facebook connections. The study’s outcome of interest is self-reported depressive disorder as published by the Centers for Disease Control and Prevention.

**Results:**

On average, 21% (21/100) of all adult residents in the United States reported a depressive disorder. Depression frequency was the lowest for counties in the Northeast (18.6%) and was highest for southern counties (22.4%). Social networks in northeastern counties involved moderately local connections (SCI 5-10 the 20th percentile for n=70, 36% of counties), whereas social networks in Midwest, southern, and western counties contained mostly local connections (SCI 1-2 the 20th percentile for n=598, 56.7%, n=401, 28.2%, and n=159, 38.4%, respectively). As the quantity and distance that social connections span (ie, SCI) increased, the prevalence of depressive disorders decreased by 0.3% (SE 0.1%) per rank.

**Conclusions:**

Social connectedness and depression showed, after adjusting for confounding factors such as income, education, cohabitation, natural resources, employment categories, accessibility, and urbanicity, that a greater social connectedness score is associated with a decreased prevalence of depression.

## Introduction

Humans fundamentally require connections to other humans. Humans have lived in social groups since the dawn of civilization [1]. The type, quality, and amount of these social connections affect psychological well-being and physical health [2]. Social connectedness decreases human mortality, improves cancer survival, cardiovascular health, and body mass, results in better-controlled glucose levels, and strengthens mental health [3].

Engaging in social media is a popular pastime for billions of people worldwide and is considered a normative means of staying connected to other people for the technological generations. Even though it purportedly offers connectedness, social media provides the least amount of happiness among the 27 leisure activities surveyed [4,5]. The social media platform with the largest number of users worldwide is Facebook (Meta) [6], where some 1 million users log in, upload 200,000+ pictures, comment on 500,000+ posts, and react with emojis to some 4M+ posts each minute [7,8]. Since 2017, researchers employed by and collaborating with Facebook have calculated and released the Social Connectedness Index (SCI) to measure the density and geographic distribution of an individual’s connections on this social media platform [9,10].

The composition of a social network, or the individuals, and groups that make up an individual’s social connections, can play a significant role in mental health. A strong and supportive social network can have a protective effect on mental health, while a lack of social connections can increase the risk of mental health problems such as depression and anxiety [11]. Interventions that focus on building and strengthening social connections, such as community-based programs or online support groups, can be particularly beneficial for individuals who have a limited social network [12]. Additionally, interventions that target specific groups, such as older adults or individuals with disabilities, who may be at a higher risk of social isolation, can also be effective in promoting mental health [13-15]. Another important consideration is the diversity of an individual’s social network. A diverse social network can have a protective effect on mental health by providing individuals with a wider range of perspectives and experiences. Understanding the composition of an individual’s social network can inform interventions and policies aimed at promoting mental health by building and strengthening social connections, targeting specific groups, and promoting diversity and inclusivity.

Studies have shown that time spent using more recently developed social media platforms (eg, Twitter and Instagram) is linked to personal insecurity and negative mental health outcomes [16]. Measures of social media network connection have shown public health utility in predicting the spread of infectious diseases (eg, COVID-19), and text analyses of social media posts have described the public’s reaction to public health messaging and interventions [17-22]. However, few public health studies have leveraged large social media data to classify user network structure and geographic reach rather than solely using social media platforms. The incorporation of composition and social connection distance allows for the exploration of how populations may maintain networks across boundaries and how these “cumulative-type” lists of connections may differ in the user-perceived social support for behavioral health given a temporal and geographic disconnect from social media friends.

The objective of this study was to determine the association between population-level web-based social connectedness and reach and depression in the population across geographies of the United States.

## Methods

### Study Design

Our study provides an ecological assessment of aggregated, cross-sectional population measures of social connectedness, and self-reported depression across all counties in the contiguous United States. We used measures obtained between 2018 and 2020 for adult residents in this area.

### Measures

The study’s outcome of interest is self-reported depressive disorder published at the county level by the Centers for Disease Control and Prevention’s PLACES project [23]. The PLACES project disseminates measures pertaining to 27 chronic diseases for all US census tracts and counties. The PLACES depression measure derives from answers to the Behavioral Risk Factor Surveillance System (BRFSS), an annual, nationally representative survey designed to aid program planning for states by providing prevalence estimates of health behaviors and outcomes. To report a depressive disorder diagnosis, a survey respondent must answer “yes” to the survey question: “Have you ever been told by a doctor, nurse, or other health professional that you had a depressive disorder (including depression, major depression, dysthymia, or minor depression)?” We used the most recently published data from 2018 to estimate the percentage of county residents with a depressive disorder.

The study’s main exposure of interest is the SCI, a pair-wise composite index describing the “strength of connectedness between 2 geographic areas as represented by Facebook friendship ties” [9]. This measure describes the density and geographic reach of county residents’ social network using Facebook friendships, and it can differentiate between local and long-distance Facebook connections. Specifically, the SCI is a measure of the probability of each Facebook user having a connection to another Facebook user across the geography of their respective residential locations, from county *i* to county *j*. We downloaded the August 2020 version of this measure from Facebook’s *Data for Good* platform [9].

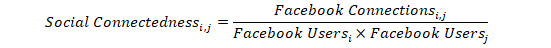



To create a measure that works as a composite, we calculated the relative probability of Facebook friendships (Social connectedness*_i,j_*) as a county-level representation of the relative probability of Facebook users in county *i* being connected to given users in county *j* (SCI*_i,j_*). The scale of the relative probability is classified using ranks of the county’s measure to categorize SCI into groups relative to the top quintile in the United States (ie, <1, 1-2, 2-3, 3-5, 5-10, 10-25, 25-100, or ≥100 20^th^ percentile). The higher the SCI rank, the more likely it is that county residents have more connections and that those connections span a larger geographical area. Lower ranks imply both fewer connections and relatively more local connections.

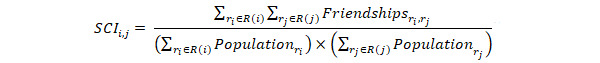



We included several covariate measures to control for confounding due to the county’s urban or rural status, natural surroundings of the area (eg, greenspace, lakes, and mountains), geographic connectivity, and population characteristics. Because urban or rural status may affect social media use or depression prevalence [24], we included a measure of county-level urbanicity using the urban or rural classification scheme of the Centers for Disease Control and Prevention National Center for Health Statistics from 2015 (eg, large metropolitan, medium metropolitan, small metropolitan, micropolitan, and noncore) [25].

As physical surroundings like beautiful scenery may affect happiness and depression [26-28], we included the Natural Amenities Scale, a measure created by the US Department of Agriculture Economic Research Service, to represent the natural surroundings of a county area that enhance that location as a place to live (eg, climate, topography, and water area) [29]. We operationalized this measure by ranking counties comparatively across the continental United States, with a rank of 1 indicating the fewest natural amenities and a rank of 7 indicating the most natural amenities.

We derived a measurement of geographic accessibility for each county from the Environmental Protection Agency’s Environmental Quality Index (EQI; 2006-2010) built environment component [30]. This measure provides a composite score describing each county’s roads, highway safety, commuting behaviors, business and housing environments, walkability, and green space. We created quintiles of the EQI to summarize the traversability of the built environments for each county, with the first quintile representing the highest accessibility and the last quintile representing the lowest accessibility.

Increasing income increases happiness, albeit with diminishing returns [31]. Educational status, age, gender, and cohabitating may further affect depression and happiness. To adjust for these factors, we collected median household income (US dollars), the average age of county residents (years), the percentage of male residents, the percentage of residents with the highest educational attainment of high school degree (including equivalency) or greater, and the percentage of cohabitating adult residents from the American Community Survey 5-year estimates from 2016 to 2020.

As chronic illness and depression are linked [32], we added a measure of self-reported poor physical health status from the BRFSS 2018 survey as a proportion of the adult population self-reporting 14 or more days during the past 30 days during which their physical health was not good.

Occupation is associated with depression through job-related psychosocial stressors and socioeconomic status [33-35], and occupation-related travel expands the geographic reach of occupational and social networks [36-38]. Therefore, we incorporated information from the American Community Survey occupation by industry tables into our analysis. We included 4 classes of occupations: (1) management, business, science, and the arts; (2) service; (3) sales and office; and (4) natural resources, construction, and maintenance.

### Statistical Analysis

Descriptive statistics of US counties and the 4 US census regions (ie, Northeast, Midwest, South, and West) were generated using frequencies, percentages, means, and SD. We used a global Moran Index test of spatial autocorrelation for each variable [39]. We identified local clusters of hot and cold spots using Anselin local Moran Index test for clustering with 9999 permutations, to which we applied a false discovery rate correction for multiple comparisons [40,41]. All independent variables were *z* score–standardized prior to modeling. Nonspatial generalized least squares regression bivariate models were fit for each independent variable with the outcome (depression). We fit a multivariable generalized least squares model to test the spatial autocorrelation of the regression residuals and the variance inflation factors. We assessed model fit via accepted and well-known specification tests and the Akaike Information Criterion [42-45]. We determined the statistical significance of measures using an α level of .05. We used ArcGIS Pro 2.9 (Environmental Systems Research Institute) to create study maps, and we conducted all analyses in RStudio (version 1.2; R Foundation) [46,47]. For the maps, we visualized major cities with over 500,000 residents. We obtained Dominic Russel’s script to process the Facebook social connectedness data for US county units through a web-based repository [48]. We implemented spatial autocorrelation and clustering tests and econometric models using the *spdep* and *spatialreg* packages [49,50].

### Ethics Approval

Our study used aggregated and anonymized publicly available data sets. Data were not linked to identifiable information to ensure privacy and confidentiality, accuracy and reliability, transparency and openness, and adhere to any applicable data usage policies. This study was reviewed, and a waiver of informed consent was granted by the University of Texas Health Science Center Institutional Review Board (HSC-SPH-22-0181).

## Results

Descriptive statistics for counties in the continental United States, stratified by census regions, show regional patterns in our outcome and main independent measures (Table 1). County-level self-reported depression was lowest for counties in the Northeast (mean 18.6% of adults) and highest for Southern counties (mean 22.4% of adults). Social networks in Northeastern counties contained moderately local connections (SCI 5-10 the 20th percentile for 79, 36% of counties), whereas social networks in Midwest, Southern, and Western counties included mostly local connections (SCI 1-2 the 20th percentile for n=598, 56.7%, 401, 28.2%, and 159, 38.4%, respectively).

Many counties (42.1/100, 42.1%) across the United States are noncore (ie, rural). The West had the most counties with high natural amenities (rank=7 for 69.3%, n=285 counties), whereas the Midwest had the most counties with low natural amenities (rank=1 for 41.5%, n=438 counties). Similarly, the West had the highest frequency of counties with the most traversable environments (EQI=1 for 42.6%, n=191 counties). The South contained the most counties with the least traversable environments (EQI=5 for 31.5%, n=448).

The Western region had the highest percentage of people working in the 4 occupation classes. In the Northeast, counties had the highest median household income (mean US $65,277, SD US $16,591). The overall average age of residents was 41.6 (SD 5.5) years, and there was an average male proportion of 50.1% (50.1/100). The Southern United States had the highest frequency of county residents reporting being in poor general health (mean 25.2%, SD 4.7%). The Midwest and the South had the highest frequency of residents with a high school education or greater (mean 34.9%, SD 6.5% and mean 34.9%, SD 7.4%, respectively). Additionally, the Midwest had the highest proportion of residents classified as cohabitating (mean 58.2%, SD 5.4%).

All predictor measures included in our study displayed significant spatial autocorrelation (Table 2, column 1), and all predictors except for the average age of county residents were significantly associated with self-reported depressive disorders (Table 2, column 2). These associations were robust to adjustments for covariates and spatial structure, except for urbanicity (Table 2, columns 3 and 4). The best-fitting model was a multivariable spatial eigenvector filter generalized least squares model (Table 2, column 4). As the quantity and distance that social connections span (ie, SCI) increases, the prevalence of depressive disorders is 0.3% (SE 0.1) lower per rank. In other words, counties classified as the most connected (ie, SCI≥100 the 20th percentile) had a depressive disorder prevalence that was 2.1% lower than counties considered the least connected (ie, SCI<1 the 20th percentile). Greater income, age, and percent of male residents (β=.1, β=.3, and β=.3, respectively) were all associated with fewer depressive disorders. Predictors showing positive associations with depressive disorders included natural amenities (β=.2, SE .0), the built environment (β=.2, SE .0), management, business, science, and the arts (β=.3, SE .1), service occupations (β=.1, SE .0), poor general health (β=.9, SE .1), and a high school education or more (β=.2, SE .0).

**Figure 1:**
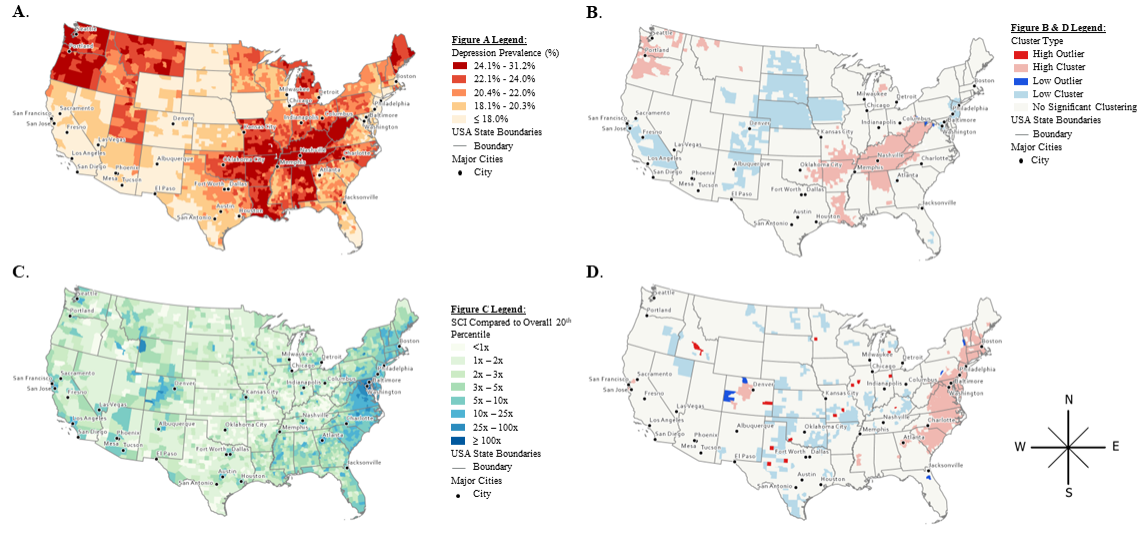
County social connectedness, depression, and local cluster measures in the continental United States. Displayed here in depression quantiles (A), local clusters of depression (B), Social Connectedness Index percentile ranks (C), and local clusters of social connectedness (D).

The geographic distribution of depressive disorders was visually clustered along the Southwestern banks of the Mississippi River, in Appalachia, and in the Pacific Northwest (Figure 1, Panel A). Our local Moran I analysis shows clusters of high (ie, “hot spots”) and low (ie, “cold spots”) depressive disorder prevalence across the contiguous United States (Panel B) that confirm the visual analysis of Panel A. Local clusters of low depression prevalence comprised the state of New Jersey and surrounding counties, a large part of the northern Midwest, central California, and southern California. The majority of Tennessee, Kentucky, and West Virginia counties are part of a local cluster of high depression, as are parts of surrounding states (eg, Alabama, Arkansas, and Louisiana) and the Pacific Northwest (Panel B). In addition to these concordant clusters, the local Moran I analysis identified areas with sharp changes in prevalence over spatially contiguous regions (ie, low and high outliers). Specifically, one county (ie, Philadelphia County, Pennsylvania) was a high outlier of depressive disorder prevalence, and 3 counties (ie, Washington County, Pennsylvania; Garrett County, Maryland; and Frederick County, Virginia) were low outliers.

**Table 1:** Descriptive statistics of county characteristics by census region (N=3142).

		Overall	Northeast^a^	Midwest^b^	South^c^	West^d^
Depression (%), mean (SD)		21.0 (3.8)	18.6 (6.4)	19.9 (3.2)	22.4 (3.2)	20.2 (3.2)
**Social Connectedness Index Percentile, n (%)**						
	<1×—Lowest social connectedness	300 (9.4)	0 (0)	132 (12.5)	124 (8.7)	36 (8.7)
	1-2×	1162 (37.4)	4 (1.8)	598 (56.7)	401 (28.2)	159 (38.4)
	2-3×	503 (16.2)	25 (11.5)	173 (16.4)	232 (16.3)	73 (17.6)
	3-5×	467 (15.0)	55 (25.4)	93 (8.8)	250 (17.6)	69 (16.7)
	5-10×	376 (12.1)	79 (36.4)	48 (4.6)	197 (13.9)	52 (12.6)
	10-25×	230 (7.4)	47 (21.7)	11 (1.0)	151 (10.6)	21 (5.1)
	25-100×	70 (2.3)	7 (3.2)	0 (0)	59 (4.2)	4 (1.0)
	≥100×—Highest social connectedness	8 (0.3)	0 (0)	0 (0)	8 (0.6)	0 (0)
**Urbanicity, n (%)**						
	Noncore	1310 (42.1)	41 (18.9)	520 (49.3)	569 (40.0)	180 (43.5)
	Micropolitan	637 (20.5)	46 (21.2)	232 (22.0)	261 (18.4)	98 (23.7)
	Small metro	355 (11.4)	27 (12.4)	112 (10.6)	167 (11.7)	49 (11.8)
	Medium metro	369 (11.9)	38 (17.5)	78 (7.4)	207 (14.6)	46 (11.1)
	Large metro	437 (14.1)	65 (30.0)	113 (10.7)	218 (15.3)	41 (9.9)
**Natural Amenities Index Rank, n (%)**						
	1—Fewest natural amenities	440 (14.3)	0 (0)	438 (41.5)	0 (0)	2 (0.5)
	2	440 (14.3)	32 (15.3)	263 (24.9)	141 (10.0)	4 (1.0)
	3	440 (14.3)	26 (12.4)	104 (9.9)	306 (21.8)	4 (1.0)
	4	440 (14.3)	50 (23.9)	67 (6.4)	312 (22.2)	11 (2.7)
	5	440 (14.3)	72 (34.5)	96 (9.1)	243 (17.3)	29 (7.1)
	6	440 (14.3)	28 (13.4)	55 (5.2)	281 (20.0)	76 (18.5)
	7—Most natural amenities	440 (14.3)	1 (0.5)	32 (3.0)	122 (8.7)	285 (69.3)
**Environmental Quality Index Quintiles, n (%)**						
	1—Highest accessibility	628 (20.0)	21 (9.7)	319 (30.2)	97 (6.8)	191 (42.6)
	2	629 (20.0)	37 (17.1)	265 (25.1)	235 (16.5)	92 (20.5)
	3	628 (20.0)	64 (29.5)	201 (19.1)	301 (21.2)	62 (13.8)
	4	629 (20.0)	71 (32.7)	164 (15.6)	341 (24.0)	53 (11.8)
	5—Lowest accessibility	628 (20.0)	24 (11.1)	106 (10.1)	448 (31.5)	50 (11.2)
**Occupation (%), mean (SD)**						
	Management, business, science, and arts	33.1 (7.0)	37.9 (7.0)	33.6 (6.0)	31.4 (7.0)	35.1 (7.2)
	Service	17.7 (3.8)	18.0 (2.7)	16.8 (3.3)	18.0 (3.9)	19.0 (4.6)
	Sales and office	20.0 (3.1)	20.4 (1.8)	19.4 (2.8)	20.4 (3.1)	19.7 (3.8)
	Natural resources, construction, and maintenance	12.5 (4.2)	9.9 (3.0)	12.1 (3.5)	12.6 (4.2)	14.0 (5.2)
Median household income (US $), mean (SD)		54,850 (14,600)	65,277 (16,591)	56,558 (10,594)	50,882 (14,973)	58,653 (16,388)
Age (years), mean (SD)		41.6 (5.5)	43.1 (4.1)	42.1 (5.2)	41.0 (5.2)	41.7 (7.2)
Male (%), mean (SD)		50.1 (2.4)	49.6 (1.9)	50.2 (1.7)	49.8 (2.9)	50.8 (2.5)
Poor general health (%), mean (SD)		21.8 (5.5)	16.8 (6.3)	19.2 (3.7)	25.2 (4.7)	19.6 (4.1)
≥High school education (%), mean (SD)		33.9 (7.4)	33.6 (8.5)	34.9 (6.5)	34.9 (7.2)	28.2 (7.0)
Cohabitation (%), mean (SD)		56.4 (6.7)	56.6 (5.2)	58.2 (5.4)	54.6 (7.4)	57.9 (6.9)

^a^Northeast census region: Pennsylvania, New York, New Jersey, Rhode Island, Connecticut, Massachusetts, New Hampshire, Vermont, and Maine.

^b^Midwest census region: North Dakota, Minnesota, Wisconsin, Michigan, South Dakota, Iowa, Illinois, Indiana, Ohio, Nebraska, Kansas, and Missouri.

^c^Southern census region: Texas, Oklahoma, Arkansas, Louisiana, Mississippi, Tennessee, Kentucky, Alabama, Georgia, Florida, West Virginia, Maryland, Delaware, Washington DC, Virginia, North Carolina, and South Carolina.

^d^Western census region: Washington, Idaho, Montana, Wyoming, Oregon, California, Nevada, Utah, Colorado, Arizona, and New Mexico.

**Table 2:** Bivariate and multivariate regressions of SCI and depression.^a^

		Unadjusted			Adjusted	
		Moran Index	Nonspatial GLM,^b^ β (SE)	Nonspatial GLM,^b^ β (SE)		Spatial filter LM,^c^ β (SE)
Social Connectedness Index Percentile		0.7^d^	−.7 (.1)^d^	−.3 (.1)^d^		−.3 (.1)^d^
Urbanicity		0.5^d^	−.3 (.1)^d^	.1 (.1)		.1 (.0)
Natural Amenities Index Rank		0.8^d^	.2 (.1)^d^	.2 (.1)^d^		.2 (.0)^e^
Environmental Quality Index Quintiles		0.5^d^	1.0 (.1)^d^	.2 (.1)^e^		.2 (.0)^d^
**Occupation (%)**						
	Management, business, science, and arts	0.4^d^	−1.2 (.1)^d^	.2 (.1)^e^		.3 (.1)^d^
	Service	0.3^d^	.3 (.1)^e^	−.0 (.1)		.1 (.0)^e^
	Sales and office	0.1^d^	.1 (.1)	.0 (.1)		.1 (.0)^e^
	Natural resources, construction, and maintenance	0.3^d^	.0 (.1)	−.9 (.1)^d^		−.0 (.0)
Median household income (US $)		0.3^d^	−1.1 (.1)^d^	−.5 (.1)^d^		−.1 (.0)^d^
Age (years)		0.3^d^	.1 (.1)	−.1 (.1)		−.3 (.0)^d^
Male (%)		0.1^d^	−.4 (.1)^d^	−.4 (.1)^d^		−.3 (.0)^d^
Poor general health (%)		0.7^d^	2.0 (.1)^d^	2.1 (.1)^d^		.9 (.1)^d^
≥High school education (%)		0.5^d^	1.4 (.1)^d^	.7 (.1)^d^		.2 (.0)^d^
Cohabitation (%)		0.3^d^	−.2 (.1)^e^	.8 (.1)^d^		.0 (.0)

^a^Results indicating lower depression associated with higher social connectedness, less natural amenities, higher built environment accessibility, more of the workforce in management, business, science, and arts, service, or sales and office occupations, higher median household income, older age, larger male population, lower proportion of population in poor general health, and higher educational attainment.

^b^GLM: generalized linear model.

^c^LM: linear model.

^d^*P*<.001.

^e^*P*<.05.

The highest-ranking category of social connectedness in the United States (ie, SCI≥100) was spatially concentrated in 1 area and comprised 8 counties: the District of Columbia; Alexandria, Arlington, Fairfax, and Falls Church counties in Virginia; and Prince George’s, Montgomery, and Charles counties in Maryland (Figure 1, Panel C). Although this map shows a clear pattern of high SCI in metropolitan counties with low SCI in their surrounding counties, the concentrated SCI area around Washington, DC, with the 8 highest-ranked SCI counties, extended far beyond the urban centers. Our local Moran I analysis indicated a cluster of high social connectedness along the Eastern coast; in this cluster, 2 counties were classified as low outliers of social connectedness (ie, Washington County, New York and Mifflin County, Pennsylvania; Panel B). Additionally, there was a cluster of high social connectedness in Colorado with 3 low outliers (ie, San Miguel County, Mesa County, and Jackson County; Panel D). In Florida, Putnam County stood out as having low SCI compared to surrounding areas (Panel D). Clusters of low social connectedness were present across states in the Midwest and Southern regions. We identified 13 counties across the United States where social connectedness was high compared to low neighboring counties, suggesting localized hubs of social connectedness that did not extend to surrounding populations (Midland, Lubbock, and Taylor counties in Texas; Jackson County, Oklahoma; Kiowa County, Colorado; Blaine County, Idaho; Stevens County, Minnesota; Boyle County, Kentucky; Pulaski and Greene counties, Missouri; Isabella County, Michigan; McDonough and Peoria counties, Illinois; and Allen County, Indiana).

## Discussion

### Principal Findings

Exploring the county-level SCI and depression showed that social connections on Facebook that span larger geographic areas are associated with fewer county residents reporting depressive disorders. This association was robust to both the type of model and adjustments for confounding factors such as income, education, cohabitation, natural resources, employment categories, accessibility, and urbanicity. Further, a visual analysis of these 2 population-based measures shows clear local and regional patterns that are robust to formal spatial analysis.

### Contextualization

We found the proportionally highest SCI value in areas where policy-making (Washington, DC) and highly localized, exclusive socialization happens (eg, Aspen, Colorado, and Jackson Hole, Wyoming). Washington, DC (policy making) and Atlanta, Georgia (public health and basic science) had higher SCI values than New York City, New York, and Los Angeles, California. To make connections across larger distances, individuals must have the means and opportunity to travel and make connections in far-flung places. The high SCI scores in counties surrounding Washington, DC, could be driven by the proportion of residents that work for, support, or lobby various government or congressional entities [51]. These individuals are likely to have many long-distance Facebook relationships with friends, family, and constituents from their home states, have frequent meetings with out-of-town connections or travel for work. Other clusters of places representing geographic hubs of high social connectedness include locations favored by US individuals with affluence. Therefore, SCI arguably measures the ability and means to travel, make novel connections, and have high levels of influence; members of these groups likely have higher social status through their income, education, and occupation. If SCI measures one’s affluence and ability to build a supportive or successful environment, then the effect on the reduction of depression may be largely influenced by access to favorable systems (eg, financial, health care, and occupational).

The absence of personal autonomy and agency has been linked to depression; thus, counties with a concentration of individuals who are not only highly autonomous but also highly influential may have lower rates of depressive disorders [52]. Further examples of this relationship can be observed in counties identified through our tests of local spatial autocorrelation. In Texas, Midland, Lubbock, and Taylor counties all share a similar vein of occupational influence and connection. These counties contain concentrations of oil, gas, wind energy, and cotton production compared to the rest of East Texas. Counties such as Taylor, Texas, Jackson, Oklahoma, and Pulaski, Missouri, all have large military bases. Lastly, Boyle County, Kentucky, has a small liberal arts college that has hosted multiple vice presidential debates and trained numerous alumni who became politicians. In addition, certain regions across the United States displayed commonly seen patterns of health outcomes: the weather in the Pacific Northwest is well-known as a cause of the seasonal affective disorder [53], and the high prevalence of depressive disorders in Southern states and Appalachian regions reflect similar patterns of poor health outcomes in diabetes, cancer, and opioid use disorders in this region.

As seen in the US map of depression prevalence, there is a notable pattern of low prevalence in certain states, such as Nebraska, North and South Dakota, Iowa, and Wyoming. This pattern is not an artifact of the BRFSS data and has been noted in prior years of collection [54]. These states are largely underserved by mental health professionals and have low availability of services, leading to artificially low rates of mental illness diagnoses [55]. In addition, these states contain large low-income populations, which may lead to low health care access and make them difficult to reach by the state-based BRFSS survey.

### Limitations

This study has limitations requiring discussion. First, this study uses areal data drawn from separate cross-sectional sources; both the ecological fallacy and the study design limit our ability to detect evidence of truly causal relationships and apply our macro-level inferences to individual-level relationships. Despite these challenges, we were able to ascertain a novel association between social connectedness and depression, and including data for all areas of the contiguous United States enabled us to provide a detailed, nationally relevant analysis of this association.

Facebook usage rates can vary depending on factors such as age, education level, and income. Additionally, some individuals may choose not to use Facebook for personal reasons, such as privacy concerns or a lack of interest in social media. Although the Facebook data were derived from all 2.74 billion users [56], providing one of the largest samples of social media networks across the United States, younger generations have left Facebook for newer platforms with different foci and sharing methods (eg, Snapchat, TikTok, and YouTube) [57]. Therefore, younger Americans are underrepresented in the measure of social connectedness.

Finally, we employed a measure of depression based on self-report, which is subject to known biases; however, these data represent the only source of publicly available, nationally representative, county-level measures of depression [58].

### Conclusions

In this study, we uncovered a previously unexplored correlation between county-level social connectedness and depression. Given the known association between the use of social media and depression—where use and interaction with social media causally increase depression—our results suggest that SCI is not a measure reflecting the use of social media but instead functionally measures social network size, affluence, and social influence. To determine if increasing a location’s SCI can be used to reduce depression prevalence, the next investigation should focus on localities that have engaged in cultural exchange programs, which increase interchanges of people and cultural activities, and controls.

### Implications for Clinical Practice or Health Policy

Social media has become a prevalent aspect of modern communication and is linked to both positive and negative effects on mental health. Therefore policy makers and community leaders need to consider the potential impact of social media use on individuals and communities and develop strategies to promote healthy use. One key aspect of social media policy making is establishing guidelines for the responsible use of social media platforms. This can include measures such as age restrictions and education on the potential risks of excessive social media use [59]. Another important consideration is the development of community-based interventions to prevent depression and other mental health issues related to social media use. These interventions can include support groups, counseling services, and educational programs that teach individuals how to use social media in a healthy and balanced way [60]. Additionally, community leaders can work with social media companies to promote positive content and provide resources for individuals who may be struggling with mental health issues. In summary, social media policy-making and community-based interventions can play an important role in promoting healthy social media use and preventing depression and other mental health issues [61]. By establishing guidelines for responsible use, addressing cyberbullying, and providing support and resources for individuals, policy makers and community leaders can help ensure that social media is used in a way that promotes well-being for all.

Major health policy developers, like the US Preventative Task Force and the US Department for Health and Human Services, have identified goals for the current decade to address access to screening and treatment of depressive disorders for the US populace [14,15]. Equitable access to mental illness diagnosis and treatment has yet to be established in the United States for all persons and geographies. Specifically, many studies of accessibility fail to consider the geographic differences in economic accessibility of mental health services (eg, transportation availability and cost, and local proximity to various providers and facility types) [62,63]. The use of technology and social networks has been suggested as a promising approach to reaching these populations in need [64,65]. New clinical approaches like telehealth and behavioral intervention technologies allow for services to reach those often underserved or isolated, whether through financial disparities or geographic isolation [66,67]. The relationship between Facebook’s SCI and the indicated relationship to social capital in the United States points toward the potential for new approaches to mental health intervention through social networking sites, through which clinicians and policy makers can target and reach populations in need [68].

## Appendix

Multimedia Appendix 1 High resolution map of county social connectedness, depression, and local cluster measures in the continental United States.

## Abbreviations

BRFSS: Behavioral Risk Factor Surveillance System
EQI: Environmental Quality Index
SCI: Social Connectedness Index
