# Correction: The Association of a Geographically Wide Social Media Network on Depression: County-Level Ecological Analysis

**DOI:** 10.2196/47896

**Published:** 2023-04-11

**Authors:** Alaina M Beauchamp, Christoph U Lehmann, Richard J Medford, Amy E Hughes

**Affiliations:** 1 Department of Epidemiology, Human Genetics, and Environmental Sciences School of Public Health University of Texas Health Science Center at Houston Dallas, TX United States; 2 Peter O'Donnell Jr School of Public Health University of Texas Southwestern Medical Center Dallas, TX United States; 3 Department of Pediatrics University of Texas Southwestern Medical Center Dallas, TX United States; 4 Lyda Hill Department of Bioinformatics University of Texas Southwestern Medical Center Dallas, TX United States; 5 Clinical Informatics Center University of Texas Southwestern Medical Center Dallas, TX United States; 6 Department of Internal Medicine University of Texas Southwestern Medical Center Dallas, TX United States

In “The Association of a Geographically Wide Social Media Network on Depression: County-Level Ecological Analysis” (J Med Internet Res 2023;25:e43623) the authors made one addition.

Multimedia Appendix 1 was added to provide a high resolution version of Figure 1 in the publication.

The correction will appear in the online version of the paper on the JMIR Publications website on April 11, 2023, together with the publication of this correction notice. Because this was made after submission to PubMed, PubMed Central, and other full-text repositories, the corrected article has also been resubmitted to those repositories.

## Appendix

Multimedia Appendix 1 High resolution map of county social connectedness, depression, and local cluster measures in the continental United States.

