# Supplementary figures and images for: The Association of a Geographically Wide Social Media Network on Depression: County-Level Ecological Analysis
Source: J Med Internet Res. 2023 Mar 27;25:e43623. doi: 10.2196/43623 (PMC10131939; doi:10.2196/43623)

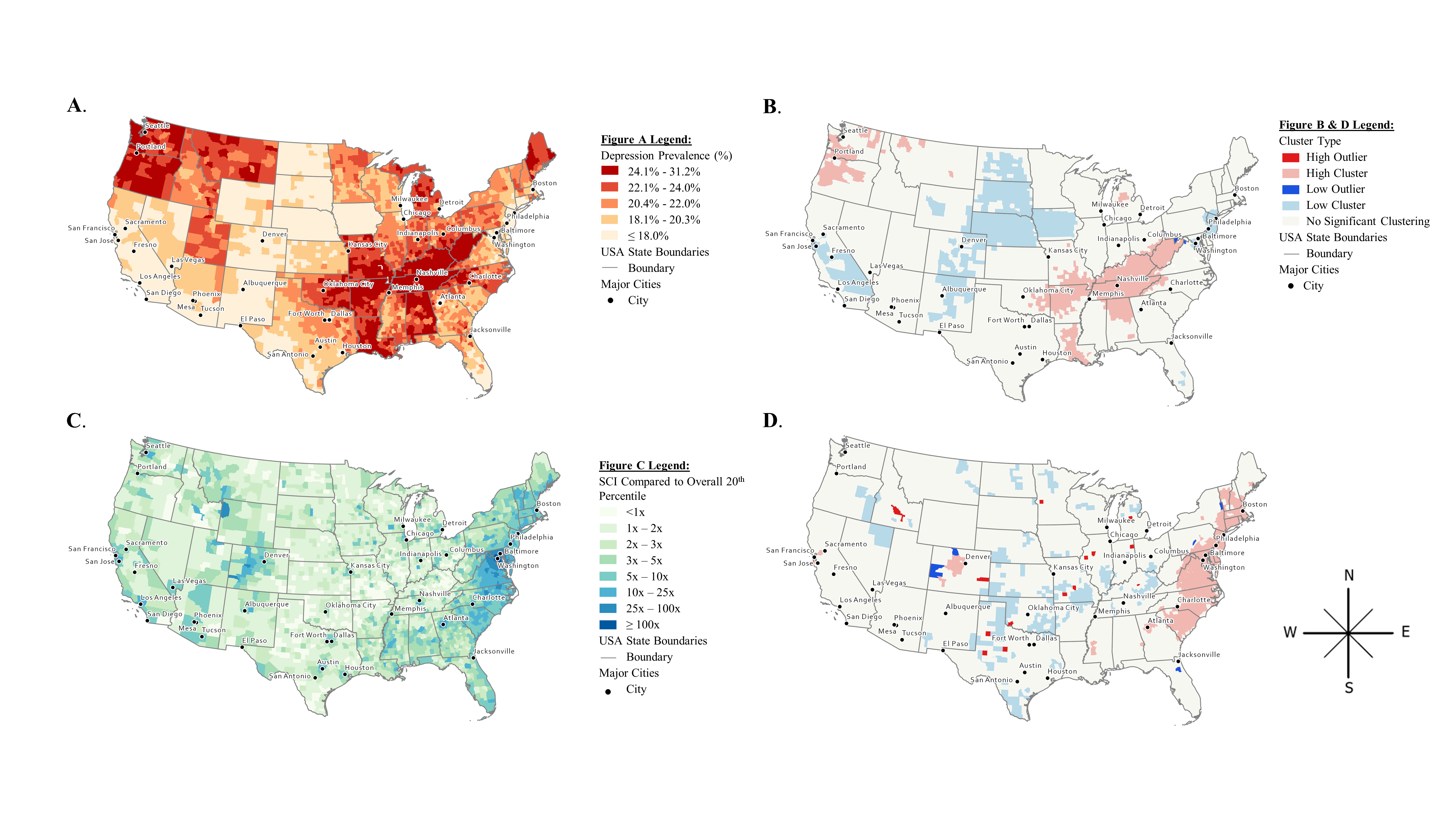

Supplement: Multimedia Appendix 1 [file jmir_v25i1e43623_app1.png]
